# Exosome-based nanoimmunotherapy targeting TAMs, a promising strategy for glioma

**DOI:** 10.1038/s41419-023-05753-9

**Published:** 2023-04-03

**Authors:** Hong Luo, Hao Zhang, Jinning Mao, Hui Cao, Yihao Tao, Guanjian Zhao, Zhiwen Zhang, Nan Zhang, Zaoqu Liu, Jian Zhang, Peng Luo, Yuguo Xia, Yuan Cheng, Zongyi Xie, Quan Cheng, Guodong Liu

**Affiliations:** 1 Department of Neurosurgery, The Second Affiliated Hospital, Chongqing Medical University, Chongqing, China; 2 Health management center, The Second Affiliated Hospital, Chongqing Medical University, Chongqing, China; 3 Brain Hospital of Hunan Province, The Second People’s Hospital of Hunan Province, Changsha, China; 4 The School of Clinical Medicine, Hunan University of Chinese Medicine, Changsha, China; 5 School of Pharmacy, Fudan University, Shanghai, China; 6 College of Life Science and Technology, Huazhong University of Science and Technology, Wuhan, China; 7 Department of Interventional Radiology, The First Affiliated Hospital of Zhengzhou, Zhengzhou, China; 8 Department of Oncology, Zhujiang Hospital, Southern Medical University, Guangzhou, China; 9 Department of Neurosurgery, Xiangya Hospital, Central South University, Changsha, China; 10 National Clinical Research Center for Geriatric Disorders, Xiangya Hospital, Central South University, Changsha, China

**Keywords:** CNS cancer, CNS cancer, Nanobiotechnology, Drug delivery

## Abstract

Exosomes, the cell-derived small extracellular vehicles, play a vital role in intracellular communication by reciprocally transporting DNA, RNA, bioactive protein, chains of glucose, and metabolites. With great potential to be developed as targeted drug carriers, cancer vaccines and noninvasive biomarkers for diagnosis, treatment response evaluation, prognosis prediction, exosomes show extensive advantages of relatively high drug loading capacity, adjustable therapeutic agents release, enhanced permeation and retention effect, striking biodegradability, excellent biocompatibility, low toxicity, etc. With the rapid progression of basic exosome research, exosome-based therapeutics are gaining increasing attention in recent years. Glioma, the standard primary central nervous system (CNS) tumor, is still up against significant challenges as current traditional therapies of surgery resection combined with radiotherapy and chemotherapy and numerous efforts into new drugs showed little clinical curative effect. The emerging immunotherapy strategy presents convincing results in many tumors and is driving researchers to exert its potential in glioma. As the crucial component of the glioma microenvironment, tumor-associated macrophages (TAMs) significantly contribute to the immunosuppressive microenvironment and strongly influence glioma progression *via* various signaling molecules, simultaneously providing new insight into therapeutic strategies. Exosomes would substantially assist the TAMs-centered treatment as drug delivery vehicles and liquid biopsy biomarkers. Here we review the current potential exosome-mediated immunotherapeutics targeting TAMs in glioma and conclude the recent investigation on the fundamental mechanisms of diversiform molecular signaling events by TAMs that promote glioma progression.

## Facts

- TAMs potentially establish the complicated, unique intercellular interactions of the glioma ecosystem. Meanwhile, they also provide a potent alternative for the targeted therapeutics.
- Exosomes would provide us with promising nanoplatforms for targeted drug delivery with enormous advantages, which would open a new era of eradication of tumors under immunoregulation.
- TAM-centered strategies can be divided into reducing the recruitment or depletion and reprograming into the M2-like phenotype.
- Exosomes would significantly assist the TAMs-centered treatment as drug delivery vehicles and liquid biopsy biomarkers.

## Question

- What is the difference between different types of macrophages, such as microglia and bone marrow-derived macrophages?
- How to realize the clinical applications of exosomes and overcome challenges such as massive production, standard isolation, drug loading, stability, quality control, etc.?
- How to design the combined modality therapy with higher efficacy?
- How to make a better transition from pre-clinical trials to clinical applications?

## Introduction

Gliomas, accounting for ~25% of primary CNS tumors, have an average mortality rate of 4.43 per 100,000 and 16,606 deaths per year [1, 2]. Standard therapies of surgery resection combined with radiotherapy and alkylating agent chemotherapy showed little curative effect, with a median survival of 16 months [3]. Intensive research and clinical efforts have revealed growing knowledge about glioma [4]. However, there has been no significant breakthrough in therapeutics [5]. Efforts like temozolomide, bevacizumab (a molecule blocking vascular endothelial growth factor-A (VEGF-A)), cilengitide (a molecule blocking αVb3 and αVb5 integrin), lomustine, etc. [6], offered minimal survival benefit during the past decades [6–8]. Therefore, more effective therapeutic options are urgently needed. Accordingly, lots of laboratory research and increasing clinical laboratory investigations have been initiated to aid standard therapies [9]. Unfortunately, up to now, the result is still far from satisfactory. Many approaches have achieved encouraging outcomes in preclinical and early clinical stages, like immune checkpoint inhibitors (ICIs), including antibodies against PD-1, its ligand PD-L1, CTLA-4, and CAR T cell therapy, failed to exert their therapeutic effects in glioma [10]. The possible reasons are various. Besides the high heterogeneity and plasticity, the key obstacles to a better treatment response mainly fall in the blood-brain barrier (BBB) and the immunosuppressive tumor microenvironment [11]. For successful therapy, effective delivery and target specificity are still essential and fundamental hamper facing us [12].

On the one hand, the BBB, which significantly impedes the transportation of most drugs, is another factor considerably limiting and restraining the treatment efficacy [13]. For better anti-tumor drug efficacy, researchers continuously strive to deliver platforms for better efficiency. The nanomaterial-based delivery platform is an alternative and versatile drug transportation system that could come promisingly across the BBB. As various nanoparticles are engineered in more specified manners, they can be modulated for therapeutic agents transporting in a more personalized way, indicating the era of precision medicine [14]. With extensive advantages like relatively high drug loading capacity, adjustable therapeutic agents release, enhanced permeation, and retention effect [15], striking biodegradability, excellent biocompatibility, and low toxicity [16], etc., exosome-based strategies is gaining increasing attention. Exosomes, a kind of cell-derived extracellular vehicles with ~30–200 nm in diameter, play a vital role in intracellular communication by transporting cell-derived proteins, lipids, glycoconjugates, nucleic acids, etc [17]. With the enormous potential to be developed into targeted drug carriers, cancer vaccines, as well as noninvasive biomarkers in diagnosis, treatment evaluation, and prognosis prediction, exosome-based therapeutics is a research hotspot in recent years [18, 19].

On the other hand, immunotherapy, a strategy aiming to control and clear tumors by modulating the immune system to regain the anti-tumor immune response and constrain the tumor escape [20], has been a hot topic in recent decades. As a booming and promising therapeutic regimen, immunotherapy shows significant clinical value in a crowd of tumors like a subset of hematological malignancies, melanoma [21, 22], and non-small-cell lung cancer [23], etc. Accordingly, accumulating efforts have been made in glioma immunotherapies. General immunosuppression in the glioma microenvironment (GME) significantly contributes to tumor progression and treatment resistance. The GME is now receiving increasing attention. Emerging research efforts are being made for an in-depth understanding of GME [24]. Grossly, the immunosuppressive microenvironment, which poses significant challenges for cancer treatment by promoting tumor progression and limiting the infiltration of immune cells, is mainly composed of extracellular matrix, soluble molecules, various brain-resident cells, and some immune cells [25]. Tumor-associated macrophages (TAMs), the ample stromal cells of the innate immune system, are a significant component of the GME that occupies 30–50% of the tumor mass [25, 26]. High TAMs infiltration is usually associated with reduced overall survival of glioblastoma [27]. CD204 + TAMs enriched glioblastoma has a different characteristic of upregulated genetic expression related to tissue hypoxia, glioma angiogenesis, and rapid invasion, favoring a poor prognosis [28]. TAMs significantly contribute to the immunosuppressive microenvironment and strongly influence glioma progression, providing us with promising targets for therapeutic strategies. Tumor immunotherapy therapies have gained tremendous advances following the rapid multidisciplinary progress, including developments in oncology, pharmacology, immunology, molecular biology, etc [29]. In addition, long-term and continuous release of immunotherapy drugs is necessary for enhancing anti-cancer immunity, and nanotechnology ensures the accumulation, controlled release, and precise release of immunotherapy drugs in tumor areas [30]. Nanoimmunotherapy, nanoparticle-based tumor immunotherapy, shows unique biological properties to achieve precise targeting, local drug delivery, enhanced therapeutic efficacy, and tremendous potential as a personalized and synergistic treatment regimen. In this review, the promising exosome-based nanoimmunotherapy targeting TAMs is a promising immunotherapy strategy that uses nano-sized exosomes as a delivery system of specific therapeutic drugs targeting the TAMs to modulate TAMs behavior, thus inhibiting the recruitment of TAMs, depleting TAMs or reprogram TAMs from the tumor-promoting phenotype to tumor-suppressing phenotype.

Exosomes, the nanosized endogenous extracellular vehicles, have colossal prospects to be potential carriers that also have great potential to stimulate an anti-glioma immune response. Simultaneously, TAMs, the significant barriers to immunotherapies that contribute to the immunosuppressive GME, also represent promising drug targets for glioma immunotherapy. This article stresses the potential application of exosome-mediated nano immunotherapies targeting TAMs. We concluded the formidable role of TAMs within the GME for glioma progression and immunosuppression *via* various molecular signaling events.

## Potential exosome-based strategies in tumor

### Brief knowledge about exosomes

Exosomes, with a small size of 30–200 nm, are cell-derived extracellular vesicles delivering specific cargo to the targeted cells. Like a double-edged sword, they have complicated functions with the potency to be either tumor-promoting or tumor-suppressing. Enriched in particular proteins, lipid bilayer, DNA, RNA, and glucose chains, exosomes regulate the extracellular matrix and transmit essential signals to other cells, affecting various aspects of cell biology [31, 32].

First, exosomes can induce signaling *via* receptor-ligand interaction, or integrate into the target cell’s membrane, thus transporting their cargo into its cytosol [33]. Then, once activated, exosomes function as modulators of the phenotypic or molecular state of the recipient cells according to their respective cargos, consequently playing an essential role in mediating intercellular communication spanning from physiological tissue regulation to pathogenic injury and organ remodeling [32, 34, 35]. For details about exosome biogenesis, content, and functions under various conditions, readers can refer to previous excellent reviews [17, 36]. Although already discovered in 1983, knowledge about the detailed mechanism of exosomes is still lacking, and the clinical application of exosomes in disease diagnosis, treatment, etc., is still challenging [26]. With molecular transfer functions and considerable immunogenicity, exosomes are widely studied for targeted drug delivery [37]. Also, interest is emerging in investigating the potential of exosomes for liquid biopsy [38].

### Vital biological function and main application potential of exosomes in glioma

**Fig. 1:**
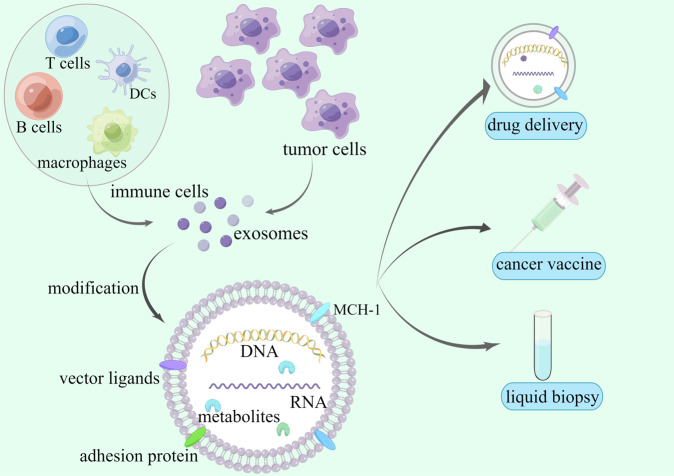
Brief structure of clinical application of exosomes. Exosomes are produced by almost all living cells, mainly immune cells (including macrophages, NK cells, dendritic cells, T cells, B cells, etc.) and tumor cells. The cargo of exosomes can be roughly divided into proteins, lipids, nucleic acids, and metabolites. The cellular membranes contain specific surface adhesion proteins, vector ligands (CD11b, CD18 receptors, integrins, glycoproteins, tetraspanins, etc.), and MHC. After specific modification, exosomes could promisingly assist tumor diagnosis and treatment as drug delivery systems, cancer vaccines, and liquid biopsy biomarkers. (By Figdraw).

Figure 1 provides a brief overview of exosome structure and potent application.

#### Exosomes as a drug delivery platform

Exosomes, the endogenous small extracellular vesicles, can transport bioactive materials to the specific recipient cells. The cargos are diverse and roughly divided into proteins, lipids, nucleic acids, and metabolites [39]. As significant intercellular vehicles, exosomes can also be used as therapeutic agents transporting platforms for specific targeting. Similar to strategies of incorporating drugs into liposomes—a synthetic spherical vesicle [40], the theory of exosome-mediated drug delivery is not new. Technologies about therapeutic drug loading, surface modification, and mass manufacture of exosomes have been comprehensively reviewed in previous publications [41]. With cellular membranes that contain specific surface adhesion proteins and vector ligands (CD11b, CD18 receptors, glycoproteins, tetraspanins, integrins, etc.), exosomes show distinct advantages of low toxicity (Naturally generated in living tissue, exosomes have the potential of preventing leakage of the drug before it reaches the targeted cells), non-immunogenicity, enhanced bioavailability (Exosomes can get the targeted cells more effortlessly *via* various mechanisms, including membrane fusion, receptor-dependent absorption, endocytosis mediated by clathrin, or *via* the clathrin-independent pathways, phagocytosis, macropinocytosis, lipid raft–dependent uptake, caveolin-dependent internalization and so on, thus possibly represent higher clinical effectiveness [32, 42]), good permeability, and specific tissue tropism, etc [43–45]. One of the major problems of many other therapeutics is the poor infiltration of the candidate drugs into the BBB. As a promising nano platform that can efficiently cross the BBB with specific targeting capacity, exosomes gain increasing attention in targeted drug delivery.

Clinically, as excellent candidates for high-quality therapeutic substance carriers, the emerging study investigates the potential of exosomes to deliver diverse therapeutic payloads, including nucleic acid (RNA, DNA, CRISPR/Cas9), chemotherapy drug (standard anticancer agents like curcumin, doxorubicin, and paclitaxel), antisense oligonucleotides, immune modulators, etc. Besides, exosomes can be engineered as a delivery platform for antibody fragments or monoclonal antibodies as a “display platform.” Those engineered exosomes act as a crux that rebuilds the interaction between cancer cells and immune cells, thus significantly promoting anti-tumor efficacy [37, 46]. Intravenous administration of unmodified exosomes from macrophages efficiently penetrates the BBB. The double-layer membranes of exosomes powerfully protect their cargos from proteases, nucleases, and other environmental impacts, thus successfully delivering specific loads into the brain [47].

The paclitaxel-containing exosomes significantly improve the anti-tumor efficacy in glioblastoma through enhanced targeting [48]. Moreover, the biocompatible macrophage-derived exosomes can be employed as carriers to deliver therapeutic agents. Myung Soo Kim et al. formulated a targeted paclitaxel delivery system by engineering macrophage-derived exosomes to load paclitaxel and incorporate aminoethylanisamide-polyethylene glycol vector moiety, which could particularly link to the sigma receptor, the overexpressed surface molecules of lung cancer cells [49]. Lydia Alvarez-Erviti et al. successfully demonstrated exosome-mediated siRNA delivery to the mouse brain [44]. J Wolfers et al. revealed that tumor-derived exosomes could carry antigens to dendritic cells, consequently stimulating the immune response [50]. Jiahui Zhang et al. reported that neutrophil-exosomes deliver cytotoxic proteins to induce tumor cell apoptosis [51]. Neutrophil-exosomes carrying the drug also show excellent chemotactic function and BBB penetration [52].

Moreover, researchers developed a dual-functional exosome-based superparamagnetic nanoparticle cluster (SMNC-EXO) using various superparamagnetic nanoparticles anchored to exosomes. SMNCEXOs can precisely deliver therapeutic agents to targeted cells in response to external magnetic fields [53].

While despite the great prospect of exosomes as competent nanosized drug-carrier, clinical applications still face obstacles like substantial production, efficient isolation, convenient therapeutic agent loading, better stability, controllable quality management, etc. Subsequently, artificial exosomes, including ‘hybrid exosomes’, ‘exosome‑mimetic’, and ‘nanovesicles’, are emerging to overcome the limitations of biological exosomes [54].

#### Exosomes as intercellular communication mediators and cancer vaccines

Originally thought to be a mechanism for eliminating cellular “waste”, exosomes are now well recognized to possess strong intercellular communication and local or distant microenvironment modulation. They are attracting accumulating attention as endogenous modulators [55]. Depending on parental cells and pathophysiological conditions, exosomes selectively package, secrete, and transfer specific molecules between cells [17]. With different origins and their respective cargos, exosomes exhibit distinct and enormous properties to change the fate of the recipient cells by modifying the translational profile in a relatively efficient, potent, and managing way [56]. Exosomes significantly facilitate intercellular communication. A further understanding of the underlying molecular mechanism would be significant for developing other anti-tumor therapeutics, including strategies targeting TAMs to reverse the immunosuppressive microenvironment.

Increasing evidence indicates that exosomes play multiple roles in cancer progression, and the dual potential of exosomes to be either cancer-promoting or suppressing has been considered. Interaction between tumor and stromal cells *via* exosomes is complex. Glioma cell-derived exosomes play crucial roles in immune modulation and can reprogram TAMs and drive polarization of macrophages to the M1 or M2 phenotype, depending on the respective molecular constituent. For example, tumor-derived exosomal miR-934 can be tumor-promoting by inducing M2 macrophage polarization. In addition, glioblastoma-derived exosomes reprogram TAMs to produce exosomes containing unique proteins that are immunosuppressive and tumor-growth-promoting [57]. Exosomal STAT3 derived from glioblastoma stem cells traverses the monocyte cytoplasm, causes a molecular change of the actin cytoskeleton, and induces monocytes to polarize toward the tumor-promoting M2 phenotype [58]. Besides, dendritic cell-derived exosomes with TNF superfamily ligands can boost tumor cell apoptosis [59], Natural killer, cell-derived exosomes with miR-186 exert cytotoxic effects on blastoma cells [60, 61]. Neutrophils-derived exosomes, with the different constitutions of a vast repertoire of cytokines, immunosuppressive or stimulatory molecules, can be pro-tumorigenic or antitumorigenic [62]. Also, glioma-derived exosomal EGFRvIII is transferred to neighboring glioma cells lacking EGFRvIII and then activates the MAPK and AKT pathway, horizontally transforming the phenotype among subsets of cancer cells [63].

More importantly, exosomes from specific sources exhibit different content profiles. For instance, the unique content of exosomes derived from tumor cells or immune cells containing a mass of tumor antigens like MHC-I could directly induces anticancer immunotherapy. With the potential to elicit antitumor responses, accumulating investigations are trying to develop exosomes into vital cancer vaccines or vaccine adjuvants [64, 65]. Exosome-based vaccines have presented infusive and hopeful results against various tumors. Exosome-based vaccines already approved have infusive and bright effects against various tumors and are approved by the FDA. TheraCys® is applied to treat early-stage bladder cancer, PROVENGE can be used for metastatic castration-resistant prostate cancer, and IMLYGIC® shows its efficacy in metastatic melanoma [66].

#### Exosomes as potential glioma biomarkers

In addition to their therapeutic potential, exosomes also present the potential to facilitate disease diagnosis and prognosis as robust biomarkers. Released continuously by all living cells, including glioma cells, exosomes are enriched in body fluids such as blood, urine, saliva, cerebrospinal fluid, sputum, etc [32, 67]. With advantages of minimal invasive characteristic, cost-saving, real-time evaluation of tumor condition, and so on, liquid biopsy attracts increasing attention in cancer. Compared with evaluating circulating tumor cells or other strategies, exosomes offer extraordinary advantages. Exosomes show high biological concentration, continuously released by alive cells, there is a relatively high level of cell-derived exosomes in various biofluid. In addition, containing different cell-derived molecules like DNA, RNA, bioactive protein, chains of glucose, metabolites, exosomes have more abundant information. And cargos contained within exosomes are protected from degradation [38, 68]. Exosome-based liquid biopsies provide information about the actions of tumor cells and present possible applications for tumor diagnosis, therapeutic response monitoring, and prognosis evaluation [55, 69]. Specifically, compared to circulating tumor cells, exosomes give better sensitivity during the initiating process, which is closely linked to carcinogenesis [70].

As shown above, exosomes act as a critical mediator of intercellular communication. Exosomes are significantly associated with tumor progression and microenvironment modulation. The exosome-based gene test to predict malignancies was the earliest available for prostate cancer in 2016 [71]. For CNS tumors, where sample collection is a chief obstacle, exosomes unfold a new vista for clinical evaluation. The structural and functional properties of exosomes make exosomes to be potent diagnostic/prognostic makers. With the potential to efficiently cross the intact BBB [72], Exosomes provide a valuable alternative that is less invasive than cerebrospinal fluid sampling. Exosomes derived from malignant glioma cells have been investigated for searching for robust biomarkers for glioma evaluation. For instance, exosomes containing significantly high MCT1 and CD147 indicate malignant glioma progression [73]. What’s more, exosome-derived miRNAs, the ample and fundamental biomolecules mediating intercellular communication, play essential roles in immunosuppression, induction, intrusion, metastasis, and treatment unresponsiveness of tumors [74]. The serum exosomal EGFRvIII mRNA of glioblastoma patients could be used to provide sufficient diagnostic information [55]. In short, many circulating exosomes and exosomal cargos that may play significant roles in the intricate cross-talk systems in glioma initiation, development, and dissemination, also provide us potent opportunities for glioma diagnosis and prognosis evaluation. To monitor drug response in GBM, Shao et al. have developed a microfluidic chip to evaluate the levels of unique exosomal mRNA (MGMT and APNG) [75]. In addition, for detecting prognostic biomarkers in glioma-derived exosomes, the TiO2-CTFE-AuNIs real-time label‐free plasmonic biosensor demonstrates its promising application in liquid glioma biopsy [76].

## The vital role of TAMs in the GME and potential strategies underlying

**Fig. 2:**
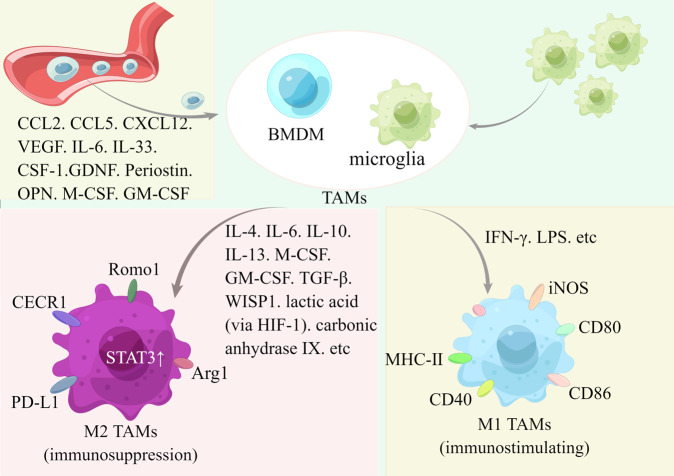
Recruitment and polarization of TAMs. TAMs consist of residual microglia and bone marrow-derived macrophages (BMDMs). Various molecules are associated with the recruitment and polarization of TAMs. Under different stimuli, macrophages show distinct phenotypes, which can be roughly divided into immunosuppressive M2 phenotype (There’s significant upregulation of STAT3, PD-L1, Arg-1, CECR1, Romo1, etc.) and immune-stimulating M1 phenotype. (By Figdraw).

The tumor microenvironment is widely recognized as critical in tumor development and treatment response. TAMs, the effective innate immune system, constitute a diminishing population among the complex components of the tumor microenvironment. In addition, they are the vital mononuclear phagocytes that bridge the natural immune response with the adaptive immune response by presenting relevant antigens to the T cells following the phagocytosis of apoptotic cells. It is not surprising that TAMs are increasingly recognized as crucial in normal processes like neural development, homeostasis, and central nervous system diseases. At the intersection of neuroscience and immunology, investigations on macrophage biology are gaining increasing attention. Meanwhile, knowledge about TAMs is fast-growing [77, 78]. (Fig. 2).

### A brief classification of TAMs

From the origin perspective, glioma TAMs consist of microglia and bone marrow-derived macrophages. Macrophages reside in most significant tissues and contribute to tissue homeostasis and disease. Following the expression of unique sets of transcriptional regulators, the progenitor cells differentiate into tissue-specific macrophages during organogenesis. Most adult macrophages, resident or infiltrating, are classical hematopoietic stem cells (HSC)-derived, except microglia. Nevertheless, the mechanisms responsible for the development of macrophage diversity remain unclear. The exact origins are still under debate [79–81]. Microglia, a population of resident macrophages derived from erythro-myeloid progenitors of the yolk sac [81], is the type of macrophages in the central nervous system under normal conditions. As a whole, microglia turn over slowly, and individual cells can potentially be decades old [82]. Besides serving as professional phagocytes, microglia significantly regulate neuronal activity at the synaptic level, ultimately supporting circuit plasticity [83]. Main classifications and more detailed information about microglia have been extensively reviewed [84, 85]. Following environmental stimuli, microglia are activated and undergo significant morphological changes to execute different tasks. Under different conditioning paradigms, the bone marrow-derived macrophages seed the brain and play a part in the parenchymal brain macrophage compartment. Nevertheless, as previously reviewed, bone marrow-derived macrophages remain distinct from yolk sac-derived host microglia in molecular and function [86, 87]. The high density of TAMs is significantly associated with poor overall glioma survival. At the same time, the distinctions between brain-resident microglia and bone marrow-derived macrophages are noteworthy. Higher infiltration of bone marrow-derived macrophages usually indicates poorer survival, while there is no such association between the quantity of microglia and the overall survival of the patient. Primarily, in glioblastoma, TAMs represent a significant population. Up to 50% of the tumor mass is macrophages. Among the total population TAMs, bone marrow-derived macrophages account for 85%, and brain-resident microglia account for the resting 15% [88].

From the function perspective, TAMs are briefly classified into an anti-tumor M1 phenotype and a pro-tumor M2 phenotype. While rather than strictly polarized M1 and M2 phenotypes, TAMs tend to be in flux between M1 and M2 states. The levels of different TAM subpopulations in the tumor core are positively associated with the overall survival of the patients [89]. With high plasticity, the traditional classifications [2] need to be reconsidered. TAMs are now well-accepted to have more complex heterogeneous subpopulations and intricate immunological functions [90]. Review of classifications of phenotypic states of TAMs has been discussed previously [84, 85, 91]. A better understanding of the complex and diverse characteristics of TAMs is essential for developing precise therapeutic strategies for glioma. While due to the limited knowledge, the oversimplified M1/M2 classification is still widely used. As the predominant part of the GME, TAMs generally express an M2 phenotype, which reflects pathognomonic features, and play a vital role in immunosuppression and glioma initiation, progression, metastasis, and treatment resistance [92].

Simply put, TAMs are recruited to the GME and then release a considerable amount of growth factors and cytokines that strongly contribute to tumor proliferation, vascularization, invasion, metabolism, treatment resistance, and the immunosuppressive microenvironment. Intriguingly, TAMs can exert duplex influences, either to orchestrate a tumor-promoting response or enhance the anti-tumor effect, which presents us with a new vista of macrophage-centered therapies [93]. As the complicated interactions establish a unique tumor ecosystem, they also offer promising opportunities for therapeutic targets with attractive prospects [94].

### Recruitment and polarization of TAMs

Under physiological conditions, exclusively *via* the IL-1α-dependent pathway, microglial populations are replenished from brain-resident microglia [95]. While under pathological conditions like trauma, infection, and brain tumor, microglia undergo substantial phenotypic changes, bone marrow-derived macrophages cross the impaired BBB and colonize the microglial niche. Distinct from other solid tumors, it is well-accepted that TAMs dominate the GME [96]. Among them, bone marrow-derived macrophages recruited peripherally constitute the significant population [94, 97]. Factors mediating TAMs chemoattraction mainly include various chemokines, surficial ligands, and other essential factors like neurotransmitters, ATP, etc [98].

TAMs are vital cells with different molecular profiles potentially regulated by microenvironment factors in sophisticated ways. Briefly, TNF-α, IFN-γ, and LPS (Toll-like receptor four ligands) drive polarization toward the M1 phenotype. While under stimulation of IL-4, IL-10, and IL-13, macrophages typically shift to the M2 phenotype [99]. At different states of differentiation, activation, and polarization, TAMs show different phenotypes and distinct functions. While regarding the separation of M1/M2 macrophages, little exclusivity was observed [93]. What’s more, as for TAMs from different sources, functions of resident microglia and recruited macrophages appear to differ in ways that remain unknown [100].

Monocyte chemoattractant protein-2 (CCL2, or MCP-1), the first identified chemoattractant molecule, is a critical molecule that recruits resident microglial cells to glioma and promotes its progression [101]. Glioblastoma cells produce kynurenine, which plays a role by stimulating aryl hydrocarbon receptor (AHR) in TAMs, which then contributes to the recruitment of TAMs *via* upregulated CCR2 expression [102]. Similarly, the up-released IL1β by TAMs activates the p38 MAPK signaling pathway and expression of CCL2 by tumor cells [103]. later, researchers confirmed a stronger correlation between the expression of CCL7 (also known as MCP-3) and the level of infiltrated microglia and macrophages [104].

CXCL12 (stroma-derived factor-1, SDF-1) is another critical chemoattraction for TAMs recruiting *via* the CXCL12/CXCR4 pathway, especially for infiltrating areas of hypoxia and tumor invasiveness [105]. Glial cell line-derived Neurotrophic Factor (GDNF) strongly induces recruiting of microglia. Accordingly, shRNA knockdown GDNF showed reduced glioma expansion and prolonged survival in mice [106].

Periostin released by glioma stem cells accumulates in the perivascular areas. It acts as a chemoattractant by stimulating the integrin receptor αvβ3 of the peripheral monocytes and M2-like TAMs [107]. Osteopontin (OPN/SPP1) also plays a vital role in recruiting macrophages to glioblastoma, regulating cellular communication between tumor cells and the TAMs *via* integrin αvβ5 on the glioblastoma-infiltrating macrophages [108]. IL-33 is associated with the recruitment and invasion of TAMs *via* platelet-derived growth factor (PDGF)–BB–PDGF receptor beta (PDGFRβ)–Sox7 signaling [109]. In PTEN-null glioma, YAP1 is activated, upregulating lysyl oxidase expression (LOX) in glioma cells. Then LOX acts as a significant macrophage chemoattractant by activating the β1 integrin-PYK2 signaling of macrophages [110].

Macrophage colony-stimulating factor (M-CSF, encoded by the CSF1 gene) significantly controls the recruitment and polarization of TAMs [111]. Tumor-derived M-CSF induces polarization of TAMs toward the pro-tumor M2 phenotype [112]. Granulocyte-macrophage colony-stimulating factor (GM-CSF, encoded by the CSF2 gene) is another essential molecule that attracts, supports survival, and induces M2 polarization of TAMs [113].

The polarization of TAMs in tumors correlates with the downregulation of the activity of the transcription agent signal transducer and activator of transcription 3 (STAT3). The enhanced levels of sialic acid of the GME, induced by the hypoxia condition, mediate the disruption of CD45 protein dimerization, upregulation of CD45 phosphatase, and the downregulation of STAT3 signaling in recruited monocytes [114]. Lactic acid expressed by tumor cells with aerobic or anaerobic glycolysis significantly promotes the expression of VEGF and the shift toward M2-like TAMs. HIF1α mediates the mechanism and the effect of lactic acid. The lactate-facilitated expression of arginase 1 by macrophages significantly promotes glioma growth [115]. Carbonic anhydrase IX contributes to the polarization of M2-like TAM through the EGFR/STAT3/HIF-1α axis under hypoxic conditions [116]. Reactive oxygen species modulator 1 (Romo1) is significantly upregulated in macrophages. The overexpression of Romo1 in macrophages induces the shift of bone marrow-derived macrophages toward M2 macrophages *via* stimulating mTORC1 signaling [117]. In addition, experiments indicate that relatively lower concentrations of S100B attenuate microglia polarization *via* inducting STAT3 [118]. Recent investigations reveal that miR-155-3p and IL-6 drive shifts toward M2 macrophages through the positive feedback loop of IL-6-pSTAT3-miR-155-3p-autophagy-pSTAT3 signaling [119].

Glioma stem cells (GSCs) release Wnt-induced signaling protein 1 (WISP1) by the signal integrin α6β1-Akt to maintain M2 TAMs. The Wnt/β-catenin-WISP1 signaling axis also presents us with a promising target [120]. GSC-derived exosomes containing various essential components, like members of the STAT3 pathway, can traverse the monocyte cytoplasm and cause the polarization of monocytes toward the M2 phenotype with upregulated expression of PD-L1 [58].

**Fig. 3:**
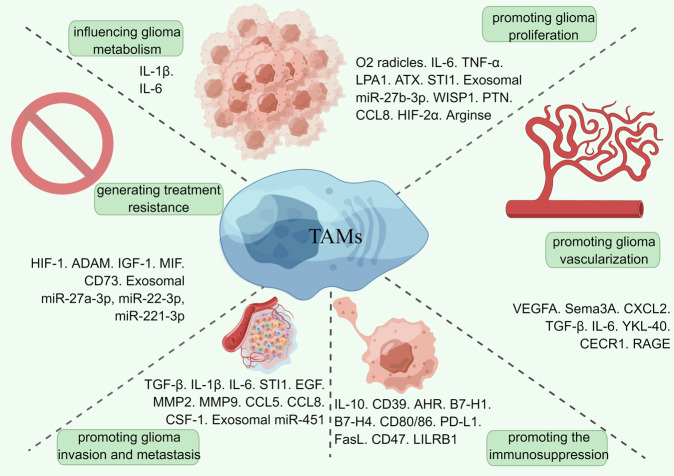
Roles of TAMs in the TME and the molecules concerned. TAMs play a significant role in glioma progression by contributing to glioma proliferation, vascularization, and invasion, promoting immunosuppression in the GME, generating treatment resistance, and influencing glioma metabolism. Acting through specific signaling pathways, various molecules are closely involved. Detailed mechanisms are discussed as follows. (By Figdraw).

Exosomal circNEIL3 can be transmitted to infiltrated TAMs, enabling them to polarize to the immunosuppressive phenotype by stabilizing IGF2BP3 [78]. ERp57/PDIA3 is another potential factor modulating microglial pro-tumor polarization toward the M2 phenotype [121]. RSK1, a downstream target of Ras/extracellular signal-regulated kinase signaling, also strongly correlates with the infiltration of M2 macrophages [122]. Also, arsenite-resistance protein-2 plays a critical role in M2-like TAM polarization *via* ARS2/MAGL signaling [123]. (Fig. 3).

### TAMs contribute to glioma proliferation

The polarized microglial cells release high levels of O2 radicals to contribute to the genomic mutations and enhance the expression of IL-6 and TNF-α to support tumor survival. TAMs play a significant role in promoting the stem-like properties of Brain tumor-initiating cells (BTICs), which possess the capacity for self-renewal and accurate recapitulation of the initial tumor and contribute to the genesis and recurrence of gliomas [124].

Microglia increase the expression of LPA1 and ATX in GBM, further contributing to GBM proliferation and migration [125]. Microglia also synthesizes and releases stress-inducible protein 1 (STI1), a cellular ligand that contributes to glioblastoma proliferation and migration.

M2 TAMs-derived exosomal miR-27b-3p raise the activity of BTICs through the MLL4/PRDM1/IL-33 signaling [126]. WISP1 also plays an indispensable role in maintaining TAMs and GSCs through the Wnt/β-catenin-WISP1 signaling [120]. TAMs secrete a large amount of pleiotrophin (PTN) to control GSCs *via* the PTN-PTPRZ1 paracrine signaling [127]. The highly expressed CCL8 on TAMs contributes to the invasive activities and stem-like characteristics of GBM cells *via* signaling on the CCR1 and CCR5 and activating the ERK1/2 phosphorylation [128].

The unreleased arginase in macrophages enhances the production of L-ornithine and putrescine, consequently playing an essential role in promoting tumor cell proliferation. Intriguingly, the metabolism of L-arginine *via* the arginase and iNOS pathways can induce distinct differences in the growth of surrounding tumor cells in association with the prevailing pathway [129]. The extracellular adenosine deaminase protein Cat Eye Syndrome Critical Region Protein 1 (CECR1), the upregulated molecule on M2 TAMs, potentially correlates with the M2-like polarization. Associated with Ki67 and mitogen-activated protein kinase (MAPK) signaling, the upregulated CECR1 significantly contributes to glioma proliferation and migration [130].

M2 macrophages secrete a large amount of IL-6, which subsequently enhances 3-phosphoinositide-dependent protein kinase 1 (PDPK1)-mediated phosphoglycerate kinase 1 (PGK1) threonine (T) 243 phosphorylation in tumor cells. This phosphorylation activity induced the PGK1-catalyzed reaction toward glycolysis by modulating substrate affinity [131]. The macrophage-regulated tumor cell metabolism mechanism implicates the prospect of a strategy to inhibit the PGK1 phosphorylation. In addition, M2 macrophages produce IL-1β, which plays a vital role in the phosphorylation of the glycolytic enzyme glycerol-3-phosphate dehydrogenase (GPD2) at threonine 10 (GPD2 pT10) through phosphatidylinositol-3-kinase-mediated activation of protein kinase-delta (PKCδ) in glioma cells. Blockade of IL-1β generation or inhibition of PKCδ, GPD2 pT10 provides us with potential treatment strategies for glioma [132].

### TAMs contribute to glioma vascularization

The rapid proliferation of tumor cells induces considerable requirement for nutrients and oxygen, leading to the neo-angiogenesis process with enhanced vascular permeability, significantly contributing to cancer progression [133]. TAMs play an indispensable role in regulating vascular homeostasis and angiogenesis of brain tumors by representing an alternative source of pro-angiogenic growth molecules. Nonetheless, angiogenesis is a complicated process involving the proliferation, migration, and differentiation of vascular endothelial cells under the activation of various signals. The well-known angiogenesis regulators in GBM progression include VEGF, angiopoietins (Angs), TGF-β, MMPs, platelet-derived growth factor (PDGF), basic fibroblast growth factor (bFGF), and hepatocyte growth factor (HGF). Besides angiogenesis, the tumor also develops a strategy of vasculogenic mimicry. In vasculogenic mimicry, the tubular structure is constituted of tumor cells, but endothelial cells efficiently transport required nutrients and red blood cells carrying oxygen to the tumor [134, 135].

VEGF is one of the significant modulators of vascular permeability and tumor angiogenesis. There is an upregulated release of VEGFA in TAMs *via* IL10/STAT3 signaling [136]. VEGFA and semaphorin 3 A (Sema 3 A) can also activate neuropilin-1 (NRP1), VEGFR1 and recruit TAMs [137]. CXCL2, the poorly described chemokine, is also significantly up-expressed and shows better angiogenic potency than VEGF in vitro [138]. The IL8/CXCL2/CXCR2 signaling axis simultaneously provides a promising therapeutical target. The AKT/mTOR signaling axis, or phosphatidylinositol 3-kinase (PI3K)/protein kinase B, is also closely associated with angiogenesis and VM formation [139].

In addition, TAMs actively control glioma angiogenesis by sensing hypoxic conditions. The enhanced levels of sialic acid in the hypoxic GME lead to a series of effects on CD45, CD45 tyrosine phosphatase, and STAT3 signaling in recruited myeloid-derived suppressor cells (MDSCs), then provide the original trigger for differentiation of MDSCs into TAMs [114].

TGF-β plays a vital role in angiogenesis’s initiation and resolution phase *via* a delicate balance of ALK5 and ALK1 signaling. The TGF-β/ALK5 signaling leads to downregulated angiogenesis, while the TGF-β/ALK1 signaling induces angiogenesis [140]. TAMs also enhance the vascular mimicry of glioma by improving IL-6 secretion in glioma cells through the PKC pathway [141]. Subsequently, JAK-STAT signaling of recruited endothelial progenitor cells are activated, promoting the vasculogenic process [142]. Also, COX2 + TAMs release IL-1β, which enhances the endothelial proliferation and upregulates the expression of pro-angiogenic regulators, including VEGF-A, hypoxia-inducible factor (HIF)-1α, and IL-8 [143].

TAMs express YKL-40 after stimulation of the MAPK–nuclear factor-kappaB (NFκB), which induces vascular endothelial cadherin/β-catenin/actin communication in endothelial cells contributing to the tumor angiogenesis process [144].

CECR1, the extracellular adenosine deaminase protein upregulated by M2 macrophages in GBM, significantly contributes to new vessel formation. Researchers confirmed that the intercellular CECR1-PDGFB-PDGFRβ signaling between macrophages and pericytes contributed to the angiogenic process [145]. RAGE (the receptor for advanced glycation end-products) signaling in TAMs significantly promotes glioma angiogenesis, providing another potential therapeutic target [146].

Moreover, an increasing number of researches indicate that resident microglia and bone marrow-derived macrophages do not show the same potential in various aspects, including vascularization of glioma. Compared with ablation of the whole myeloid cell fraction, selective reduction of resident microglia represents the equivalent effect of decreased vessel density, indicating that resident microglia, peripherally derived macrophages, are the vital modulatory cell population [147]. While current research and knowledge about microglia are still lacking, in-depth investigations into the distinct role of microglia in glioma are of essential significance.

### TAMs contribute to glioma invasion

Glioma-associated macrophages have been revealed to express several significant modulators that induce glioma growth and invasion, including IL-1β, TGF-β, IL-6, epidermal growth factor (EGF), and STI1 [148], by modulating the perivascular GICs and glioma cells [149].

Besides, TAMs also support glioma invasion by upregulated expression and activation of extracellular matrix-degrading proteases like matrix metalloprotease (MMP) 2, MMP9, and membrane-type 1 MMP (MT1-MMP) [150]. Versican released by glioma can trigger the expression of TLR2 and educate microglia into the M2 phenotype, which presents upregulated MT1-MMP expression [151]. Then, the inactive pro-form MMP becomes activated after cleaving under stimulation of MT1-MMP [152]. TGF-β, predominantly released from TAMs, can induce the up-expression of MMP and down-regulation of tissue inhibitor of metalloproteinases (TIMP)-2, thus significantly glioma invasiveness and migratory responses. Using plasmid-transcribed small hairpin RNAs (shRNAs) to suppress the expression of TGF-β type II receptor (TbetaIIR), the invasiveness and tumorigenicity were successfully inhibited in vitro [153]. CCL5 is associated with the upregulation of MMP2, thus contributing to the invasion and migration of glioma. Under the stimulation of CCL5, glioma cells subsequently increase intracellular calcium levels, phosphorylated Ca2+/calmodulin-dependent protein kinase II (p-CaMKII), and p-Akt expression levels. Not surprisingly, the levels of p-CaMKII strongly correlate with MMP2 regulation [154]. CCL8, which TAMs highly express, contributes to the invasion and stem-like characteristics of GBM cells. *Via* CCR1 and CCR5, CCL8 activates ERK1/2 phosphorylation in GBM cells and induces pseudopodia formation [128].

### TAMs generate glioma treatment resistance

Glioma, especially GBM, tends to be treatment resistant. Proneural-to-mesenchymal transition (PMT) of glioma cells is a usual mechanism for increased radiotherapy resistance. The mesenchyme differentiation induces radio-resistance in glioblastoma, associated with high infiltration of GAMs and stimulation of the TNF/NF-κb signaling axis [155]. TAMs enhance the process of PMT in GSCs through exosomal miR-22-3p, miR-221-3p, andmiR-27a-3p, which target chromodomain helicase DNA-binding protein 7 (CHD7) and regulate maintenance and development of neural stem cell, thus generate the radio-resistance [156]. A disintegrin and metalloprotease that are upregulated in GBM also contribute to chemotherapy resistance and recurrence after TMZ therapy [157].

Under CSF-1R inhibition, the tumor microenvironment drives treatment resistance *via* stimulation of the PI3K signaling caused by tumor cell IGF-1 receptor (IGF-1R) and macrophage-derived insulin-like growth factor-1 (IGF-1). A combination of IGF-1R/PI3K disturbance and CSF-1R blockade would be a potential therapeutic approach [158]. Resistance under anti-angiogenic therapies like an antibody that neutralizes VEGF (bevacizumab) is associated with the migration inhibitory factor (MIF). Blocking and downregulation of MIF induced by bevacizumab efficiently limit the M1 polarization of macrophages [159].

Immune checkpoint therapies of anti-PD-1/PD-L1 and anti-CTLA-4 show limited efficacy. Researchers have discovered a unique population of CD73^hi^ macrophages in GBM after treatment of anti-PD-1, which may be the critical mechanism of TAMs-generated treatment resistance. Respectively, under-treatment of anti-PD-1 and anti-CTLA-4, the CD73 negative murine model of GBM showed improved survival, indicating that CD73 might be another novel therapeutic target [160].

### TAMs contribute to the immunosuppressive microenvironment

Glioma represents an immunosuppressive immune environment with a low number of infiltrated lymphocytes and other types of immune effector cells [12]. In the GME, there is a high concentration of the classic immunosuppressive cytokines like TGFβ and IL-10 released by brain stromal cells, large amounts of indoleamine 2,3-dioxygenase 1 and tryptophan 2,3-dioxygenase 2 (IDO/TDO) that could both stimulate the accumulation of regulatory T cells (Treg) and suppressed T cell activity by depleting tryptophan from the microenvironment. The intercellular communication between TAMs and other types of immune cells in the TME provides an added mechanism of immunosuppression. Chemokines recruit Tregs from TAMs, then secrete IL-10, which interacts with IFN-γ expression and reduces infiltration of T cells, creating a positive feedback loop [161].

AHR enhances KLF4 expression and suppresses NF-κB signaling in TAMs. The ectonucleotidase CD39 of TAMs is subsequently upregulated, in cooperation with CD73, and promotes CD8 + T cell dysfunction *via* enhanced expression of adenosine [102]. Also, the IDO/TDO induced tryptophan metabolite L-Kynurenine (Kyn) interacts with AHR to drive the immunosuppressive effects *via* Tregs, myeloid-derived suppressive cells, and up-regulated PD-1 on CD8 + T cells [162].

GBM-initiating cells induce mTOR expression in microglia of mouse models, and the mTOR-mediated signaling of STAT3 and NF-κB contributes to the M2-like microglial phenotype, which hinders infiltration of effector T-cells, tumor proliferation, and immune responses [163]. IL6-activated STAT3 acts on the promoter of the B7-H4 gene and enhances the expression of B7-H4 on TAMs, which is an essential pro-tumor step of blocking effective T-cell immune responses [164].

TAMs also upregulate a variety of surface molecules that inhibit activation of T cells and even induce the apoptosis of T cells, including CD80/CD86 (interacts with CTLA-4 on T cells), CD95, CD70, and programmed cell death ligand 1 (PD-L1, which binds to PD-L1 on Tregs), Fas linkage (interacts with Fas receptor on CD8 + T cells). The upregulated B7-H1 expression on TAMs through IL-10 signaling contributes to the immunosuppressive phenotype [165]. Consequently, leading to a lower level of infiltrated immune effector cells and hindering the immune response against the glioma. Genetically, MYC significantly controls the expression of CD47, the critical regulator of the innate immune, and PD-L1, the adaptive immune checkpoint [166].

β2-microglobulin (β2M), the standard component of MHC-I, is expressed by cancer cells and directly protects cancer cells from phagocytosis. Binding to the leukocyte immunoglobulins (LILRB1) on TAMs, this signaling pathway is associated with inhibiting phagocytosis by depriving immune surveillance. Disrupting MHC-1 or LILRB1 signaling would enhance the process of cancer cell phagocytosis [167].

## Exosomes as drug delivery platform

It is well recognized that TAMs play vital roles in glioma proliferation, vascularization, invasion, metabolic change, treatment resistance, and immunosuppression. Basic investigations provide many TAM-targeted therapies as potential alternatives for GBM immunotherapies. Currently, TAM-targeted medicines are mainly classified into two strategies: (1) inhibiting the recruitment or reducing the population of TAMs, and (2) reprogramming TAMs from the immunosuppressive M2 phenotype to the immunostimulating M1 phenotype. The current challenges of TAMs-targeting therapeutics include the poor distribution of drugs and systemic side effects.

While traditional methods to deliver targeted therapy, such as nanoparticles and liposomes, show limited benefits due to the relatively poor bioactivity, histocompatibility, and tumor selectivity [168]. As novel drug delivery technologies, exosomes offer excellent prospects with lower toxicity, better tissue tolerance, biocompatibility, and unique lipid bilayer to confirm biological stability. Recently the carefully-designed small extracellular vesicles with trans-activator of transcription peptides and angiopep-2 possess dual delivery of targeted drugs and good cell-penetrating functions, efficiently cross the BBB, reach and penetrate the glioma niche [169]. In a word, the utilization of exosomes would undoubtedly represent a distinct and competent strategy for drug delivery, providing an encouraging vista for the treatment of glioma.

**Fig. 4:**
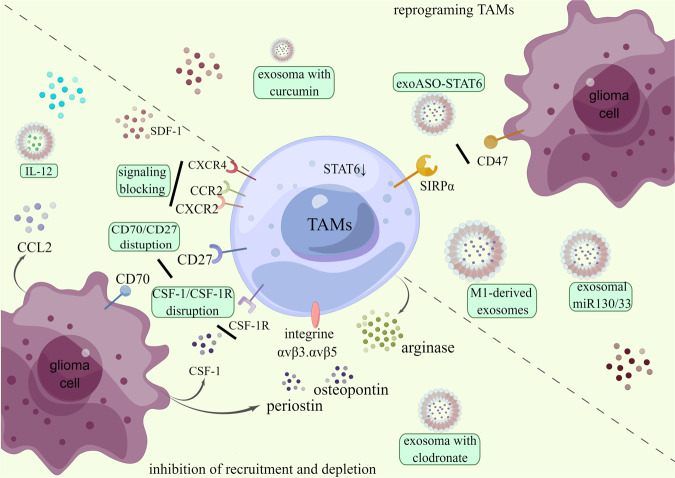
Current exosome-mediated nano immunotherapies are targeting TAMs. TAMs-targeted therapies are roughly divided into inhibiting recruitment or radically depleting TAMs from the quantity perspective and reprogramming TAMs from the quality standpoint. Exosome presents us with an encouraging nano platform for targeted drug delivery. Glioma cells are the significant group of cells inducing TAMs recruitment. There are emerging approaches striking to block or disrupt various signaling pathways between TAMs and glioma cells, such as the IL-12, CCL2/CCR2, SDF-1/CXCR4, CD70/CD27, CXCR2, CSF-1/CSF-1R, periostin/integrine αvβ3, osteopontin/integrine αvβ5 signaling. Exosomes loaded with clodronate show considerable effects on depleting TAMs. They block the CD47/SIRPα signaling *via* exoASO-STAT6 incudes upregulated STAT6, thus reprogramming TAMs to the anti-tumor M1 phenotype. Besides, exosomal miR130, exosomal miR-33, exosome loaded with curcumin, and M1 macrophage-derived exosomes also significantly re-educate TAMs. Detailed information and additional promising target are discussed as follows. (By Figdraw).

Targeting the molecular factors involved in the TAMs interaction, such as factors/receptors that modulate the phenotype of TAMs toward a proinflammatory behavior, is promising. In addition, the communication between TAMs and other cells can be performed through EVs like exosomes. Exploiting the natural ability of the exosomes as transporters, it is realizable to use them as drug delivery strategies, thus targeting the TAMs [170]. Actually, both natural and modified exosomes could offer us the platform to deliver therapeutic immunology components targeting TAMs. Modified exosomes with specific cargos, such as tumor drugs and targeting siRNA, provide us with new vista of tumor therapeutics (Fig. 4).

In brief, exosome-based nanoimmunotherapy targeting TAMs can be achieved by inhibiting the recruitment of TAMs, depleting the number of TAMs, and reprograming TAMs into the tumor-suppressing phenotype.

There is a successful practice of exosome-based therapeutics targeting TAMs. Signal transducer and activator of transcription 6 (STAT6), the key transcription factors controlling the M2 phenotype, have been “undruggable” selectively in TAMs. Administration of the engineered exosome delivering an antisense oligonucleotide (ASO) targeting STAT6 (exoASO-STAT6), which could selectively silence STAT6 expression in TAMs, induces reprogramming of TAMs and remodeling of the tumor microenvironment [171]. In addition, exosomes own specific tropism ability. The lipid bilayer and the expression of several surface molecules like prostaglandin F2 receptor negative regulator (PTGFRN) could enhance the targeted delivery [172]. For instance, M1-derived exosomes modified with IL4RPep-1 (a peptide binding to IL4R of TAMs) on the surface are more efficient than untargeted and control peptide-labeled exosomes on reprogramming TAMs into M1-like macrophage [173].

### Inhibition of recruitment and reduction of TAMs

The majority of TAMs are recruited peripherally. Inhibition of the recruitment process and strategies to deplete TAMs significantly contribute to glioma suppression. To inhibit the recruitment of macrophages into the GME, a common approach is inhibiting signaling activities associated with macrophages’ recruitment, differentiation, and survival. A variety of TAM-targeting agents are under investigation in early-phase clinical trials. Incorporating these TAM-targeted agents into exosomes would probably present a promising result.

The CCL2-CCR2 chemo-attractive signaling pathway provides an alternative target for TAMs recruitment blocking. Clinical efforts such as CCL2 or CCR2 blockade and CCL2 downregulation are underway [174]. The well-designed lipid nanoparticles loaded with CCR2-silencing siRNA blocked the monocyte recruitment and the consequent generation of TAMs, encouragingly promoting tumor regression [175]. In addition, CXCR2 antagonization (SB225002) inhibits glioma growth during tumor initiation and progression and presents a significantly decreased infiltration of TAM in recurrent tumors. The CXCR2/CXCL2 signaling represents a promising therapeutic target in glioma. Encouragingly, Combi-therapy of TMZ and SB25002 also shows an enhanced anti-tumor effect in a mouse model [176]. Combining radiotherapy with AMD3100, the clinically approved agent that inhibits SDF-1/CXCR4 interactions and blocks the infiltration of Tie-2+ monocytes, may present better outcomes [177]. Other CXCR4 inhibitors like peptide R [178], LY2510924, and plerixafor [179], are under clinical investigation with promising prospects.

CD70, a TNF family member on tumor cells but macrophages, is strongly linked to the infiltration of CD163^+^ macrophages *via* its receptor, CD27, on the cell surface [180]. The CD70/CD27 axis may be a viable therapeutic avenue. Developing exosomes containing CD70-blocking molecules may present an excellent curative effect.

Besides, other investigated TAMs-centered therapeutic targets may present us with a new vista on the exosome-mediated immunotherapies of glioma. Periostin is an exciting target for astricting the M2 TAMs by disturbing the integrin αvβ3 signaling [107]. Disruption of these signaling pathways may give us an encouraging outcome. Cilengitide, which inhibits signaling of the αvβ3 and αvβ5, is under investigation in clinical trials. While as for the recurrent GBM, it’s worthwhile to search for more therapeutic strategies like integrating cilengitide into combinatorial regimens [181]. Utilization of exosomes may give us a different result.

More radicle strategies for depleting TAMs are also under investigation. Several molecules and mAbs targeting the CSF-1/CSF-1R signaling axis are in clinical development as monotherapy and combined therapy. Inhibitors of the (CSF-1R) (anti-CSF1R antibody-like Cabiralizumab, SNDX-6352, BLZ945, PLX3397, etc.) to diminish the TAMs population in mouse GBM model significantly increases the survival and shrinks the established tumors [182]. While further preclinical and clinical trials failed to show their effectiveness [183]. Glioma may acquire resistance driven by elevating levels of high IGF-1R and macrophage-derived IGF-1, which enhance survival and invasion of glioma cells [158]. Rational combination therapies are currently promising strategies under investigation. Incorporating these therapeutic molecules with exosomes would be a direction worthy of trying.

MiR-142-3p is revealed to play a unique part in modulating M2 TAMs through the TGF-β signaling. M2 macrophages have a lower level of miR-142-3p expression compared with M1 macrophages. Therapeutic administration of miR-142-3p coherently induces M2- apoptosis and results in glioma growth inhibition [184]. Intracerebral administration of liposome-encapsulated clodronate shows promising efficacy by inducing microglial apoptosis once phagocytosed by macrophages. At the same time, a lack of specificity for TAMs causes lesions of other brain cells and damages blood vessel integrity [185]. In this respect, an exosome-based specific delivery system may present a promising prospect for TAMs-targeted therapies.

Yuan Qian et al. have developed M2-like TAM dual-targeting nanoparticles (M2NPs), the lipid nanoparticles modified with a fusion peptide composed of α-peptide (a scavenger receptor B type 1 targeting peptide) and linked with M2pep (an M2 macrophage binding peptide). Loaded with siRNA targeting anti-CSF-1R to inhibit the survival of M2 TAMs, these nanoparticles could lead to selective depletion, tumor regression, and prolong the survival of B16 melanoma-bearing mice [186]. The dual-targeting capacity of M2NPs, combined with RNA interference, provides new insight into designing TAMs-targeted therapeutics *via* exosomes.

Similarly, targeting the overexpressed folate receptors on TAMs, Tingting Luo et al. synthesized the oxygen/paclitaxel-loaded microbubbles. Combined with ultrasound-mediated delivery, these microbubbles act as immunomodulatory agents, efficiently depleting the TAMs [187].

However, there is also dispute that there are better strategies than indiscriminately diminishing the TAMs, as TAMs might play diverse roles according to the GME [188]. Although most evidence indicates a pro-tumor role of TAMs and prompts the application of TAM reprogramming therapeutics, some unique exceptions exist. In Sonic Hedgehog-medulloblastoma, researchers revealed a surprising group of anti-tumor TAMs. In these cases, TAMs deletion would instead accelerate tumor growth [189].

### Reprogram of TAMs

Reprogramming M2 to M1 macrophages has been acknowledged as a promising therapeutic strategy with better efficiency than the depletion of TAMs or immune checkpoint inhibitors. Thus, rebuilding the TAMs landscape gains increasing attention. Various approaches can achieve it, blocking different surface molecules involved in the immunosuppressive profile of TAMs like PD1-PDL1, CD206, CD63, CD204, SIGLEC1, MARCO, TREM2, etc. Inhibiting the “do not eat me” signaling to promote phagocytosis or disturbing the epigenetic activities of pro-tumor TAMs such as prostaglandin (PGE2) signaling, PI3k-γ signaling, or modulating the histone deacetylases, etc [190]. Simply put, TAMs reprogramming can be realized by inhibiting the tumor-promoting activities and stimulating the anti-tumor properties.

Exosomes, the novel nano platform for drug delivery, possess tremendous advantages in biomedical areas. Increasing attention has been paid to exosomes to exert their potential to deliver therapeutic agents. As a novel and promising strategy, the exosome-based delivery of specific modulators for TAMs reprogramming has been investigated in many malignancies. The well-designed delivery system derived from bone marrow mesenchymal stem cells, loaded with galectin-9 siRNA, and superficially modified with oxaliplatin prodrug (the molecule triggers immunogenic cell death), significantly induces tumor-suppressive macrophage polarization, achieves considerable therapeutic efficacy in pancreatic ductal adenocarcinoma [191]. Exertions have been tried in designing exosomes to deliver various therapeutic agents to re-educate TAMs, such as siRNA, chemokines, cytokines, bisphosphonate, and TLR agonists.

As aforementioned, exosomal miRNAs regulate TAMs polarization into the pro-tumor M2 phenotype. Reciprocally, alternation in levels of related exosomes may induce repolarization of TAMs toward the M1 phenotype. Exosomes rich in miR-33 and miR-130 increased the expression of M1 signature genes (IRF5, MCP1, CD80) and secretion of cytokines (IL-1β and TNF-α) as well as yeast phagocytic activity of macrophages, cheerfully decrease tumor progression by shifting M2 macrophages to M1 macrophages, providing us with a potential alternative for tumors [192]. Genetically blocking molecules involved in macrophage polarization (such as STAT3, STAT6, or homodimers of the NK-κB subunit) would result in the regulation of macrophage polarization and activation of specific immunity [193]. Chlorogenic acids (CHA) have been viewed as a potent molecule that promotes the shift of M2 TAMs into the M1 phenotype by stimulating STAT1 and inhibiting STAT6. STAT3 inhibition by siRNA or CPA-7 also shows its potential to reprogram TAMs and inhibit glioma growth in mice [194]. A nanoparticles-based delivery of mRNAs encoding interferon regulatory factor 5 and its activating kinase IKKβ promisingly reprogram TAMs into M1-like phenotype, consequently inducing anti-tumor immunity and promoting tumor regression in vitro. Apart from targeted specificity, this immunotherapy is also safe for repeated dosing [195].

Disturbance of the SIRPα-CD47 signaling provides a promising target for TAMs reprogramming. In response to CD47 blockade, microglia, and macrophages show different morphological and transcriptional changes. Intriguingly, microglia are effectively re-educated to phenotype with the unleashed potential of tumor cell phagocytosis [196]. Currently, IBI 188 and SRF-231 are the monoclonal antibodies ongoing Phase I trials testing. The M1-derived, azide-modified exosomes are designed with conjugation of dibenzocyclooctyne-modified antibodies of CD47 and SIRPα. After systemic administration, they specifically and actively recognize CD47 on the tumor cell surface and simultaneously block SIRPα and CD47 on macrophages. This led to the abolition of the “do not eat me” signal and enhanced phagocytosis of macrophages. Meanwhile, M1-derived exosomes significantly re-educate TAMs from M2 to the M1 phenotype [197]. This engineering strategy provides an exosomal platform to load modified ligands targeting TAMs.

Inspiringly, the M1-derived exosomes transfected with miR-511-3p, NF-κB p50 siRNA, and modified with IL4RPep-1 (binding to IL4R of TAMs) on the surface efficiently inhibit tumor growth by downregulating target genes, decreasing the levels of M2 cytokines and immune-suppressive cells, while increasing the levels of M1 cytokines and immune-stimulatory cells, thus repolarizing TAMs into anti-tumor M1 phenotype [173]. What’s more, the complex intercellular communication between TAMs and tumor cells critically contributes to glioma progression. Glioblastoma-derived exosomes (GBex) repolarize TAMs into M2 phenotype, and subsequently, TAMs reprogrammed by GBex produce arginase-1+ exosomes. Selectively inhibiting arginase-1 by nor-NOHA significantly reversed the tumor-promoting effects [57]. Exosomes derived from M1 macrophages have potential to repolarize M2 macrophages to M1 macrophages. Researchers also reveal that exosome-mimetic nanovesicles derived from M1 macrophages (M1NVs) potentially modulate M2 macrophages to shift toward the M1 phenotype by regulating miRNA and mRNA expression profiles. Co-injection of M1NVs and PD-L1 induces the re-education of M2 TAMs into the M1 phenotype, resulting in the inhibition of glioma in mice models [198].

In addition, it’s reported that the dietary agent curcumin, even at a low, transient concentration level, profoundly plays a distinct role in TAM repolarizing into a tumoricidal M1 state. *Via* induction of STAT-1, curcumin stimulates the M2 to M1 switch and recruitment of NK cells and Tc cells to eliminate the tumor in animal models of glioblastoma [199]. What’s more, the safety and efficacy of exosomes as curcumin carriers have been evaluated for the clinical treatment of colorectal cancer [65]. Similarly, several non-cancer-related FDA-approved drugs like antibiotic thiostrepton, iron supplement ferumoxytol, and vitamin B3, have demonstrated an incredible ability to stimulate a TAM anti-tumor activity, indicating the potential to be repurposed as adjunctive treatments [200].

Apart from the technologies shown above, many agents could be loaded into exosomes with excellent properties to present us with better therapeutic results. Programmed cell death protein 1 (PD-1), the essential immune checkpoint receptor upregulated on T cells to induce immune tolerance, is also highly expressed on TAMs. Disturbing the PD-1/PD-L1 axis directly affects the activation of TAMs [201]. A phase III trial of nivolumab (inhibitor of PD-1) in GBM patients showed comparable results and the safety profile with other tumor types [202].

Inhibitors and monoclonal antibodies that target TAMs mediated/secreted angiogenic molecules like VEGFC (VGX-100), Sema 4D, etc., show encouraging results in limiting cancer progression and reducing angiogenesis [203]. In a mouse GBM model, dual inhibition of Ang2/VEGF alters TAMs into an antitumor M1 phenotype and contributes to vascular normalization and tumor regression [204].

Amphotericin B (AmpB) acts as a stimulant of monocytoid cells and enhances the microglial effect by influencing the activities of genes involved in BTIC cycle growth arrest and differentiation, thus overcoming the tumor immunosuppressive microenvironment and curbing tumorigenicity [205]. AmpB treatments, however, have substantial toxicity.

Inhibition of CSF-1R (BLZ945) shows the potential to limit glioma progression by repolarizing TAMs into M1 phenotype in mouse GBM models. While in preclinical trials, clinical trials failed to demonstrate effectiveness, partly due to the complicated actions of non-TAMs cells in response to the agents [206].

Similarly, let-7 miRNA serves as essential TLR7 signaling stimulants that modulate the multiple functions of microglia in both healthy brains and glioma *via* the different expressions of specific sequences [207]. Previous research shows that miR-340-5p overexpression restrains TAMs recruitment (miR-340-5p targets the POSTN, thus recruiting TAMs *via* integrin αvβ3), M2 macrophages polarization (targeting LTBP-1) and tumorigenesis of glioma. The miR-340-5p feedback loop regulates the tumor microenvironment and GBM progression, presenting us novel therapeutic strategy [208].

The mannosylated nanoparticles encapsulated with siRNAs targeting the NF-κB signaling pathways show good selectivity in targeting TAMs *via* the mannose receptor (CD206), successfully inducing cytotoxic and immunostimulatory activities of TAMs in vitro [209].

TLR9 could bind with the synthetic unmethylated cytosine-phosphoguanine (CpG), which subsequently stimulates NF-κβ and AP-1 signaling and promotes macrophages to express diverse pro-inflammatory cytokines like IL-6, type I IFN, and IL-1β [210]. Encapsulating CpG onto nanoparticles may contribute to macrophage uptake and act as immunostimulating modulators. For instance, the self-assembled CpG-shell gold nanoparticles powerfully modulate macrophage polarization *via* a TLR9-dependent manner [211].

Stimulators of interferon genes (STING) agonists are another potential group of immunotherapeutic drugs that regulates innate immunity and potentially modulates the polarization of TAMs. Delivering STING agonists *via* exosomes may present us with superior therapeutic efficacy. Indeed, researchers have designed the 2′3′-cGAMP loaded nanoparticles, which efficiently enhanced STING signaling in the TME and converted TME from an immunosuppressive to an immunostimulatory one. Administration of STING-activating nanoparticles strongly remodels the tumor immune microenvironment, induces potent limitation of tumor growth, prolongs the overall survival, and improves the response of immune checkpoint inhibitors [212, 213].

In addition, exosomes may present us with a favorable alternative to deliver cytokines and innate immune adjuvants to re-educate TAMs. Similarly, researchers have synthesized microenvironment-responsive nanoparticles which could effectively administrate and release IL-12 into the tumor microenvironment, and the responsive actions could subsequently repolarize TAMs [214]. Marimastat, a famous mimetic molecule inhibiting the MMP family, is investigated in clinical trials as a promising therapeutic agent for GBM. At the same time, the result was far from satisfactory for the nonnegligible toxic side effects [215, 216]. Besides, mTOR inhibition result entirely prevents glioma-induced M2 polarization of microglial cells and increases their cytotoxic potential [217].

In addition, there is a close relationship between the reprogramming of metabolic traits and the polarization of TAMs. In the tumor microenvironment, TAMs predominantly promote oxidative glucose metabolism *via* aerobic glycolysis. Inhibiting the aerobic glycolysis of TAMs can shift M2 TAMs to M1 TAMs, contributing to tumor regression. Various factors are reported to take part in regulating macrophage metabolism [218].

In general, therapeutic strategies meet significant hurdles of substantial toxicity, poor and targetless delivery, etc. Exosomes would promisingly present better therapeutic effects as strong drug carriers. As aforementioned, therapeutic agents that could disturb the functioning of TAMs are worth investigating.

## Exosomes as potential biomarkers of TAM states

Besides drug delivery platforms targeting TAMs, exosomes can act as robust biomarkers for glioma diagnosis, therapeutic response monitoring, and prognosis evaluation. Developing new combinatory diagnostic tools in clinical practice to track TAM activation states in the brain is needed. Acting as prognostic indicators of disease outcome and monitoring new therapeutic strategies aimed at quelling a progressive pro-tumor response, exosomes would present us with encouraging vista for glioma. With profound advantages of convenient access, abundant quantity and information, biocompatible stability, and potential to cross the BBB, exosomal molecules provide an ideal alternative as glioma biomarkers [219].

Recent research indicates that circNEIL3 is related to YAP1 signaling activation and CCL2 and LOX secretion, thus driving the infiltration of macrophages. Exosomal circNEIL3 could be transmitted to infiltrating TAMs, thereby enabling them to acquire pro-tumor functionality by stabilizing IGF2BP3 [78]. Kristan E van der Vos et al. revealed that high levels of exosomal miR-451/miR-21 were transferred from glioma cells to microglia, which increased microglia proliferation and shifting of cytokine profile toward glioma promotion [220]. Mingyu Qian et al. discovered that by targeting TERF2IP to activate the STAT3 signaling pathway and inhibit the NF-κB signaling pathway, miR-1246 mediated M2 macrophage polarization [221]. MiR-1246 in the CSF might present us a novel biomarker for glioma diagnosis. Moreover, therapeutics targeting microRNA-1246 may assist the anti-glioma immunotherapy. Erik R. Abels et al. confirmed that glioma cells could reprogram microglia in part by transferring exosomal miR-21 [222], which also opens up opportunities for therapeutics aiming at disrupting this form of communication between glioma cells and TAMs. A recent study indicated that exosomal the long noncoding RNA (lncRNA) TMZ-associated lncRNA in GBM recurrence (lnc-TALC) could be delivered to TAMs and then promote M2 polarization of the microglia [223]. Liangyi Zhu et al. lately demonstrated that downregulated exosomal let-7i-5p and miR-221-3p could trigger M2 polarization of TAMs-through upregulating peroxisome proliferator-activated receptor gamma [224].

Nevertheless, the specific mechanisms by which exosome signals regulate TAMs in gliomas still need to be defined. Understanding the intricate interaction between gliomas and TAMs could open a new vista for the therapy. The utilization of exosomes as clinical biomarkers still demands further evaluation and investigation. The main requirements are validation in larger patient cohorts, more standardized methodologies for identifying exosomal biomarkers, better-isolating exosomes, more accurate quantifying of miRNAs or proteins, etc. With increasing research efforts on these applications of exosomes and continuous technological advances, diagnostic applications of exosomes in glioma will be promising shortly.

## Conclusions, challenges, and perspectives

For glioma, conventional therapies provide unsatisfactory efficacy. Most therapeutic attempts to incorporate immune therapeutics have been futile, mainly due to BBB, the complex GME, the heterogeneity of glioma tissues, off-target effects, and low immunogenicity. It is relatively challenging to “awaken” the immune activities in the complicated GME. To overcome these obstacles and achieve better efficacy, some potential directions are worth investigating: (1) develop a drug delivery platform that could efficiently cross BBB, (2) identify the complicated signaling intracellular or intercellular for developing new drugs, (3) find convincing biomarkers for more timely and precise glioma diagnosis and monitoring.

TAMs, the significant component of GME that exert nonnegligible effects on glioma proliferation, vascularization, invasion, metabolism, and treatment resistance and significantly contribute to immunosuppressive GME, is closely associated with the genetic phenotype of glioma and primarily influence the treatment response, prognosis of glioma. As TAMs potentially establish the complicated, unique intercellular interactions of the glioma ecosystem, they also provide a potent alternative for the targeted therapeutics. Indeed, various potential targets have already been identified and evaluated by further trials. Increasing attention has been paid to disrupting the specific signaling pathway using diverse interventions—nucleic acids, gene therapy, small molecule inhibitors, antagonistic or agonistic antibodies, synthetic molecules, etc. Nanoimmunotherapy targeting TAMs, the ideal weapons for precision and personalized medicine, opens a new era of eradication of tumors under immunoregulation. At the same time, the targeted delivery of therapeutic agents is still challenging to achieve and represents a significant obstacle that limits cancer treatment results. Currently, exosomes are studied as promising nanoplatforms for drug delivery with enormous advantages, including low toxicity, enhanced bioavailability, good permeability, and specific tissue tropism. More than nanosized extracellular vehicles, exosomes also act as essential regulators of intercellular communication, indicating the vast potential of exosomes to assist the diagnosis and treatment of glioma as less-invasive, real-time liquid biopsy biomarkers, which could provide a promising application for diagnosis and simultaneous monitoring. Advances in the exosomes field and a more profound understanding of the underlying function of exosomes would significantly lead to breakthroughs in clinical applications to benefit patients. Engineering the novel therapeutic exosomes, combined with TAM-targeted immunotherapy, may herald a new era of cancer immunotherapy that potentially brings opportunities to overcome existing limitations.

Deeper investigations and further understanding of the intercellular interactions between TAMs and various tumor cells and other cells in glioma would conclusively yield new glioma treatment strategies. Given that TAMs might be the potent target to facilitate immunotherapeutic efficacy, multiple directions have been investigated for qualitatively repolarizing macrophages toward the anti-tumor subtype. Besides, reducing the amounts of TAMs by inhibiting the recruitment of glioma or radically depleting TAMs also provided us with alternative methods. Microglia and bone marrow-derived macrophages show different potentials in various aspects of glioma development. Knowledge about distinctions between microglia and bone marrow-derived macrophages still needs to be improved. Further investigations into different types of macrophages are required.

The tumor microenvironment restrains the diffusion of nano-drugs, thus decreasing the therapeutic effectiveness. Martin et al. propose that nanomedicines should integrate anti-tumor agents and factors that “normalize” the diverse composition of the tumor microenvironment, inducing increased cancer perfusion and decreased levels of hypoxia. These efforts may ease drug delivery and transform the microenvironment from immunosuppressive to immunostimulating [225]. In another aspect, further investigation to realize a stimuli-responsive release of immunomodulatory therapeutic agents may present us with better efficacy.

In conclusion, TAMs significantly contribute to glioma progression *via* various signaling molecules, providing new insight into therapeutic strategies. The TAMs-centered strategies can be mainly divided into reducing recruitment or depleting TAMs and reprograming the M1-like phenotype into M2-like. Exosomes would significantly assist the TAMs-centered treatment as drug delivery vehicles and liquid biopsy biomarkers. And much work remains to be done to push forward the hopeful progress established in preliminary work. This would be crucial for developing different therapies that re-modulate the tumor microenvironment to benefit patients with glioma. With more profound knowledge of TAMs and exosomes, exosome-mediated nanoimmunotherapy may transform glioma immunotherapy in the future. We hope this review contributes to deeper investigations to advance the current understanding and continue challenging the status quo of standard glioma therapy to improve its clinical efficacy.

### Overcoming challenges of clinical application of exosomes

Exosomes could transmit therapeutic factors to the targeted cells for therapeutic applications. While there is a necessary condition that exosomes need be able to find their target and release the cargo specifically. At present, the molecular mechanisms of the related exosomes remain undefined. More basic research to better understand these unknowns would aid diagnosis and therapy of glioma shortly.

Besides, clinical applications of exosomes face several challenges, such as massive production, standard isolation, drug loading, stability, and quality control. During the past decade, efforts on basic exosome research, while challenges still exist for the therapeutic delivery of drugs via exosomes [226]. Ivano Luigi Colao et al. reviewed the present manufacturing technologies and strategies that may aid the clinical production of exosomes [227]. Important technologies available for exosome isolation have been precisely discussed by Sergio Ayala-Mar et al. [228]. For the preservation of exosomes, present data indicate that −80 °C remains the promising mode. While considering cost and poses challenges in transportation, alternatives such as lyophilization and the incorporation of additives may be needed [229]. The heterogeneity of exosomes poses a big challenge for the isolation, suitable methods to profile exosome heterogeneity have previously been reviewed [230, 231].

Artificial nanocarriers are the novel direction of drug delivery systems in nanomedicine [54]. Efforts and advances in exosome-related technologies are simultaneous and of significant value.

### Designing rational combined modality therapy

Considering the complex intercellular communication in the GBM, employing one effective single drug seems unrealistic. The high loading capacity and rich surface modification characteristics of exosomes provide us with possibilities for improving therapeutic agents’ biocompatibility and targeting capacity. Potently to load multiple drugs simultaneously, exosomes can enhance the efficacy of drugs. Engineering multifunctional exosomes is a step toward efficient immunotherapy and may even replace the current and traditional therapeutic strategies. At the same time, a deeper understanding of the complex intercellular communication between the immune system and cancer cells is required to further increase therapeutic agents’ efficacy. Targeting molecular signatures identified in the glioma context and combining several levels of TAM functionality, from genetic to epigenetic to metabolic molecules, would be a promising direction for TAM-targeted therapeutics.

### Transition from pre-clinical trials to clinical applications

Although some strategies have shown considerable therapeutic efficacy in preclinical trials, no single agent has succeeded in clinical trials. The transition from pre-clinical trials to clinical applications is still difficult. The heterogeneity and complexity of human glioma, which induces the discrepancy between patients’ responses under immunotherapies, cannot be entirely simulated by animal models or cell lines. Some may result in unexpected human side effects that animal models cannot affect. In short, more laboratory and clinical efforts are still required.

Therapeutic approaches of targeting, more specifically, the disease-associated microglia and TAM subsets while preserving the homeostatic ones provide us with a grand vista for glioma [232]. TAMs, including microglia and these bone marrow-derived macrophages, reveal diversified phenotypic identity and function, thus resulting in a continuum of states responding to environmental signals. Future studies may focus on sex differences, how bone marrow-derived macrophage subsets interact with resident microglia to regulate their phenotypic state, and whether this cross-talk can be re-directed to control the expression of pro- and anti-tumor responses. Surprisingly, the host microbiome produces some crucial signals that influence microglia. Identifying those signals and to exploiting them therapeutically is prospective [233]. In addition, identifying the pro-resolving TAMs subsets that are long-lived in the brain under disease conditions [91] may provide us with a novel direction for glioma.

Anna Gieryng et al. reviewed the glioma-derived signals and mechanisms driving bone marrow-derived macrophage accumulation and reprogramming [234]. Several glioma-derived factors are revealed to trigger the migration, proliferation, and reprogramming of TAMs. Many preclinical analyses focus on the interaction of TAMs and glioma cells, while the communication between TAMs and other immune cells is still poorly understood [87]. Much remains unclear about the complex cell-cell interactions in the glioma microenvironment. In a clinical setting, it is crucial to figure out the cellular and extracellular matrix-dependent relationships unique to the glioma microenvironment, identify potential targets involved in the intricate intracellular communication, and then find the exceptional opportunities of therapeutic approaches, hopefully contributing in the future to a better outcome for glioma [234].
